# Implementation of infection control in health facilities in Arua district, Uganda: a cross-sectional study

**DOI:** 10.1186/s12879-015-0999-4

**Published:** 2015-07-14

**Authors:** Peter Wasswa, Christine K. Nalwadda, Esther Buregyeya, Sheba N. Gitta, Patrick Anguzu, Fred Nuwaha

**Affiliations:** African Field Epidemiology Network, Kampala, Uganda; School of Public Health, Makerere University College of Health Sciences, Kampala, Uganda; Arua District Health Office, Arua, Uganda

**Keywords:** Infection control, Implementation, Health facilities, Hand washing, Healthcare workers, Uganda

## Abstract

**Background:**

At least 1.4 million people are affected globally by nosocomial infections at any one time, the vast majority of these occurring in low-income countries. Most of these infections can be prevented by adopting inexpensive infection prevention and control measures such as hand washing. We assessed the implementation of infection control in health facilities and determined predictors of hand washing among healthcare workers (HCWs) in Arua district, Uganda.

**Methods:**

We interviewed 202 HCWs that included 186 randomly selected and 16 purposively selected key informants in this cross-sectional study. We also conducted observations in 32 health facilities for compliance with infection control measures and availability of relevant supplies for their implementation. Quantitative data underwent descriptive analysis and multiple logistic regressions at 95 % confidence interval while qualitative data was coded and thematically analysed.

**Results:**

Most respondents (95/186, 51 %) were aware of at least six of the eight major infection control measures assessed. Most facilities (93.8 %, 30/32) lacked infection control committees and adequate supplies or equipment for infection control. Respondents were more likely to wash their hands if they had prior training on infection control (AOR = 2.71, 95 % CI: 1.03–7.16), had obtained at least 11 years of formal education (AOR = 3.30, 95 % CI: 1.44–7.54) and had reported to have acquired a nosocomial infection (AOR = 2.84, 95 % CI: 1.03–7.84).

**Conclusions:**

Healthcare workers are more likely to wash their hands if they have ever suffered from a nosocomial infection, received in-service training on infection control, were educated beyond ordinary level, or knew hand washing as one of the infection control measures. The Uganda Ministry of Health should provide regular in-service training in infection control measures and adequate necessary materials.

**Electronic supplementary material:**

The online version of this article (doi:10.1186/s12879-015-0999-4) contains supplementary material, which is available to authorized users.

## Background

The World Health Organization (WHO) estimates that over 1.4 million people suffer from nosocomial infections at any one time, with the proportion of these infections being up to 20 times higher in low and middle income countries [1]. These infections are among the leading cause of death and morbidity among hospitalized patients and present a considerable public health burden [2].

Although there is limited data on nosocomial infections in Sub-Saharan Africa, several studies done in Algeria, Burkina Faso, Senegal and Tanzania have indicated hospital-wide prevalence rates ranging from 2.5 % to 14.8 % [3–6]. Higher cumulative incidence rates have been reported in surgical wards in Ethiopia and Nigeria ranging from 5.7–45.8 % [7, 8]. In developing countries, a growing proportion of nosocomial infections can be assigned to methicillin-resistant *S. aureus* (MRSA) and multi-drug resistant Gram-negative bacteria [9, 10]. A survey done in an Argentinean general hospital revealed incidence rates of *Clostridium difficile,* the commonest cause of nosocomial infectious diarrhoea to range from 37 to 84 cases per 10,000 admissions between 2000 and 2005 while the annual incidence of the same infection was 8.7 cases/10 000 hospitalisations in a study done in South Africa [11, 12].

Most nosocomial infections can be prevented with readily available and inexpensive strategies like adhering to recommended infection control measures such as hand hygiene and wearing of gloves [2]. Globally, standard precautions of infection control are considered an effective means of protecting healthcare workers, patients and the public and reducing nosocomial infections [13, 14]. A meta-analysis by Aiello and Larson indicated that appropriate hand hygiene practices significantly reduced the risk of nosocomial infections while a case-control study conducted in Brazil singled-out poor hand hygiene in addition to overcrowding and understaffing as risk factors for nosocomial infections [15, 16].

A number of factors may influence adherence to infection control. A healthcare worker was more likely to be compliant if he/she had more experience on the job, was more knowledgeable about transmission of blood-borne pathogens and was strongly committed to a positive occupational safety climate [17]. A descriptive exploratory study conducted in Botswana amongst emergency department nurses identified resource constraints such as the lack of the necessary facilities, inadequate equipment and materials, inadequate staffing and the lack of sustainable in-service education as factors that could prevent them from complying with infection control measures [18].

Several studies conducted amongst doctors and nurses in Ethiopia, Nigeria, Thailand and Uganda concluded that the knowledge, understanding and interpretation of infection control measures are not adequate. This as a result adversely affected the implementation of the measures [19–22]. Although knowledge of standard precautions of infection control may improve adherence to the measures, other influencing factors which this study was not able to investigate such as attitude are equally important [21].

In Uganda, the Ministry of Health (MOH) lists five basic standard precaution measures that can enhance infection control within the health facilities. These are: hand hygiene, adequate protective wear, proper sterilization, proper sharps disposal and safe waste management [23]. However, findings from a national service provision assessment survey conducted by MOH showed that only 6 % of health facilities had all infection control items while supervisory visits to health facilities in Arua District in 2006 revealed that less than 60 % of the assessed facilities implemented the required infection control measures [24].

We assessed the implementation of infection control in health facilities in Arua district and determined the predictors of hand washing among healthcare workers.

## Methods

### Study design and setting

We conducted a health facility-based cross-sectional study in Arua district, Uganda in 2008. Arua is located about 530 km northwest of Kampala City bordering the Democratic Republic of Congo (DRC). The district had a projected mid-2007 population of about 500,000 people, 23 % of which were less than five years old [25]. There were 36 government health facilities, five private-not-for profit (PNFP) health facilities and 17 private health clinics in the district.

### Sample size determination

The formula for survey sampling by Leslie Kish was used to determine the number of healthcare workers selected for the study, where; *p* = 0.48 (estimated prevalence of health workers who wash their hands [26, 27]. The level of precision was 0.05 and confidence interval at 95 % give a sample size of 384 respondents. However, because Arua had an estimated total of 360 healthcare workers, fewer than the computed sample size of 384 respondents, Cochran’s correction formula was employed to get the final sample size of 186 respondents [28].

### Sampling procedure

We used two sampling methods to select the health facilities. Simple random sampling was used to select the lower level health units (Clinics, Health Centre (HC) II and HC III) while purposive sampling was used to select the higher level health units (both hospitals and all three HCIVs). The latter method was used in order to avoid excluding the higher level units since they performed major operations such as caesarean sections which were more likely to expose patients and health care workers (HCWs) to nosocomial infections. Using the fish-bowl technique and sampling without replacement, we randomly selected 10 out of the 12 HC IIIs in the district, 9 out of the 17 private clinics and 8 out of the 25 HC IIs. The number of healthcare workers obtained from each HC level was arrived at using probability proportionate-to-size sampling in an approximate ratio of 5: 1: 2: 1: 1 for hospitals, HCIVs, HCIIIs, HCIIs and private clinics respectively since the units were estimated to have 150, 40, 75, 50 and 50 staff respectively.

Using the list of the HCWs in each facility as the sampling frame, we selected the respondents from each health facility by simple random sampling, again adopting the fish bowl technique and sampling without replacement making sure that HCWs were sampled from all the available sections in that facility where possible. Sixteen key informants who were senior nursing officers, heads of infection control committees or health facility heads were purposively sampled from 50 % (16/32) of the health facilities that were randomly selected. All respondents who were approached for the quantitative data consented to be interviewed while one key informant who had been selected was not available during the data collection stage and was later replaced by his deputy.

### Eligibility criteria

All healthcare workers in Arua district working in the selected health facilities at the time of the study and provided written informed consent were eligible. Any health workers not present during the data collection period were excluded from the study.

### Study variables

Washing of hands before conducting aseptic procedures was the primary dependent variable. Other secondary dependent variables assessed were wearing of gloves before conducting aseptic procedures, disposal of used sharps, disposal of waste, isolation of patients and cleaning of the health facilities. The independent variables included age, sex, in-service training (number of times they had received formal training on infection control in the last five years), qualification of healthcare worker, ownership of health facility (private or government) and knowledge of infection control measures. Knowledge was assessed by determining how many of the eight major infection control measures as described by WHO [2] In table below were mentioned by the HCWs.

**Fig. 1:**
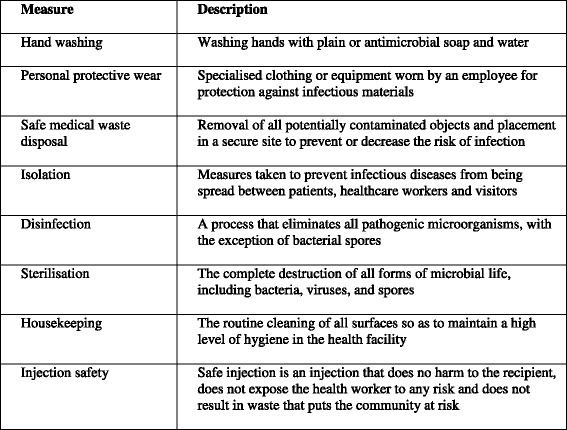
Description of infection control measures that were assessed during the study

Description of infection control measures that were assessed during the study are described in Fig. 1.

A scoring criterion of poor, fair, good and excellent adapted from Suchitra *et al.* [29] was used if a HCW mentioned 1-2, 3-4, 5-6 and 7-8 numbers of the measures respectively.

### Data collection

We interviewed HCWs using a structured questionnaire and key informants using a key informant guide (Additional file 1). The former tool was administered to the HCWs to obtain information about knowledge and practices on infection control and their responses were filled in by the data collectors while the latter tool was used to assess availability of infection control resources and capacity to monitor nosocomial infections (Additional file 1). Specific health facility sections such as injection and dressing rooms, examination rooms, laboratory and maternity wards were also observed for compliance with infection control and availability of enabling supplies such as water, soap, personal protective wear and waste disposal bins using a checklist. To ensure data quality, a team of three data collectors were trained on how to conduct the interviews and inconspicuously observe the HCWs for hand washing in 30 min intervals. The data collectors focused their observations on the five key moments recommended by WHO when HCWs should wash their hands: before patient contact, before an aseptic procedure, following exposure to bodily fluid, following patient contact, and after contact with patient surroundings [30]. The questionnaires were pre-tested in two non-participating private health clinics. Data were checked for completeness before the data collectors left the field.

### Data management and analysis

Quantitative data underwent descriptive analysis and multiple logistic regressions at 95 % confidence interval (CI). The data were first entered into an *Epi info* version 3.4.3 database and cleaned, cross-checked and then exported to *STATA,* version 10 software for analysis. For continuous variables, mean (SD) and median (range) were calculated while for categorical variables, proportions were used. Our primary dependent variable was washing of hands during the five key moments as recommended by WHO. Pearson chi-square test or Fisher’s exact test were used when appropriate in bivariate analysis. Multivariate backwards stepwise logistic regression analysis was performed to identify predictors of hand washing. All variables significant during bivariate analysis at a *p*-value ≤0.1 were introduced in the regression model to obtain the adjusted odds ratio (AOR) of each factor on the primary dependent variable at 95 % confidence interval. Qualitative data was analysed through manifest content analysis with the aid of Microsoft Excel. We read through the recorded transcripts and coded the responses. The codes were then grouped into related categories and then into emerging themes and subthemes. The main themes were capacity of the health facilities to monitor hospital-acquired infections and availability of resources to implement infection control measures. Picking responses from the themes, we used representative quotes to summarise our key findings.

### Ethical considerations

Ethical review and approval was sought from the Uganda National Council of Science and Technology through Makerere University School of Public Health Higher Degrees Research and Ethics Committee. The District Health Officers (DHOs) as well as the heads of the selected health facilities were contacted for their permission before data were collected. Written informed consent was obtained from all the respondents prior to the conduct of the interviews. Confidentially was observed and the respondents’ right to withdraw from the study without any repercussions on their work was emphasised.

## Results

### Questionnaire findings

#### Socio-demographic characteristics of the healthcare workers

The mean (SD) age of the 186 HCWs was 36.3 (8.9) years and median (range) age was 35.0 (22–59) years. The average time spent working in the health service was 11.9 (SD 8.8) years. Nearly all of the respondents (98.9 %, 184/186) had attained at least ordinary level education (11 years of formal education) as shown in Table 1.

**Table 1:** Socio-demographic characteristics of the healthcare workers interviewed on infection control in Arua district, 2008

Characteristic	Frequency *N* = 186	Percent
Age group (Years)		
20–29	45	24.2 %
30–39	83	44.6 %
40–49	39	21.0 %
50–59	19	10.2 %
Sex		
Male	85	45.7 %
Female	101	54.3 %
Highest education level		
Primary (≥7 years formal education)	2	1.1 %
Ordinary level (≥11 years of formal education)	98	52.7 %
Advanced level/ tertiary	86	46.3 %
Qualification		
Nursing Assistant	40	21.5 %
Enrolled Nurse	38	20.4 %
Registered Nurse	33	17.7 %
Enrolled Midwife	18	9.7 %
Clinical Officer	15	8.1 %
Laboratory Assistant	14	7.5 %
Registered Midwife	13	7.0 %
Others	15	8.1 %
Years in service		
0–9	88	47.3 %
10–19	62	33.3 %
20–29	24	12.9 %
>30	12	6.5 %

#### Knowledge of infection control measures

Most HCWs (51.1 %, 95/186) mentioned at least six of the eight major infection control measures we assessed while only 47.8 % (89/186) and 17.2 % (32/186) cited housekeeping and isolation respectively. Slightly over half of the respondents (51.4 %, 95/186) had ever read the Uganda Ministry of Health guidelines on infection control and only 43.5 %, (81/186) said they always followed them although this could not be verified. Most of the respondents (72.6 %, 135/186) said they had never had any in-service training on infection control, a finding which was corroborated by the key informants.

### Findings from key informants

#### Theme: capacity of the health units to monitor hospital-acquired infections

Most of the key informants reported lack of specific structures to promote infection control activities within the health facilities, contrary to what was in the MOH guidelines.

*‘We do not have committees or any structure to monitor for those infections that may be got from our health facility. However, we suspect that some infections may be acquired from poorly done surgeries at times.’* (Medical Officer)

*“For instance, when there is post-operative sepsis, we investigate where it is coming from. In February this year, we had at least three cases of post-operative sepsis in maternity”.* (Hospital Key informant)

#### Theme: availability of supplies to implement infection control measures

Stock outs were a relatively common occurrence as confirmed by most key informants. All government run health facilities reported to routinely experience some form of shortage of supplies essential for infection control during the past year. The most commonly inadequate supplies were gloves, disinfectants and soap. Whereas most stock-outs lasted about 1–2 weeks, some lasted for up to three months.

*In April, we run out of syringes and gloves for about 2 weeks. We therefore told the patients to go and buy their own supplies.* (Senior Nursing Officer)

Conversely, unlike government facilities, key informants from private facilities did not report any stock outs of infection control supplies.

#### Theme: capacity of healthcare workers to implement infection control measures

The key informants from both public and private hospitals mentioned that the health facilities were grossly understaffed, with the latter reportedly operating with only about 50 % of the expected number of healthcare staff. Likewise, remote facilities also had a significant shortage of staff. However, two health facilities in Arua municipality were over-staffed.

*“The person responsible for infection control is a registered nurse who was sent for training.*” (HCIV Key informant)

Most of the 16 key informants interviewed mentioned that they did provide regular in-service training on infection control to HCWs in their respective facilities. In most instances, all that was done were occasional reminders from the medical doctors or senior nurses about the need to implement infection control measures.

*‘We occasionally remind our staff about maintaining hygiene but this is general and not specific to infection control’* (Senior Nursing Officer)

### Checklist results

#### Observation of infection control measures

Hand washing was practiced by 74.7 % (139/186) of the health workers observed during any of the five key moments recommended by WHO while two-thirds (124/186) wore gloves when appropriate. Isolation was observed in only 6.3 % (2/32) of the health facilities while disposal of sharps in suitable containers (90.6 %, 29/32) was the most commonly observed practice. Recapping of needles (34.4 %, 11/32) was the least observed practice (Table 2). While most facilities (59.4 % 19/32) lacked functional placenta pits, the majority of the injection and dressing rooms (75 %, 18/24), were relatively clean, having no litter or significant amounts of bio hazardous waste on the operating tables or floors.

**Table 2:** Observation of infection control in health facilities in Arua district, 2008

Activity	Frequency (%)	Total N
All needles not recapped after use	11 (34.4)	32
Needles disposed in suitable containers	29 (90.6)	32
Placed sharp and non-sharp waste in separate bins	30 (93.8)	32
Sections below adequately clean^a^		
Injection / dressing	24 (75.0)	32
Examination/ consultation	21 (80.8)	26^b^
Laboratory	18 (90.0)	20^b^
Maternity	12 (75.0)	16^b^

^a^ No visible presence of blood, or any potentially infectious contaminated waste material such as used cotton, sharps or gloves on the operating table or floors

^b^ Sections not available in these facilities

#### Availability of supplies needed for infection control

All the health facilities were observed for the necessary supplies needed to implement infection control measures. The most available item was running water (90.4 %, 29/32), while the least available items were alcohol hand rubs (3.1 %, 1/32) and protective eye wear (6.3 %, 2/32) as summarised in the Table 3. Medical waste was indiscriminately disposed in all observed facilities, with sharps frequently mixed with other waste at the final rubbish dumps.

**Table 3:** Availability of infection control supplies in health facilities in Arua district, 2008

Supply	Frequency (Yes)	Percent	Total N
Hand rubs	1	3.1	32
Protective eye wear	2	6.3	32
Safety signs for hazardous wastes	6	18.8	32
Face masks	11	34.4	32
Functional placenta pit	13	40.6	32
Containers with secure lids	14	43.8	32
Gowns/aprons	16	50.0	32
Functional autoclave	16	50.0	32
Waste pit	18	56.3	32
Soap	22	68.8	32
Auto-destruct syringes	23	71.9	32
Pit latrine			
Functional	25	78.1	32
Clean	11	44.0	25
Ordinary single-use syringes	26	81.3	32
Disposable gloves	27	84.4	32
Sharps disposal containers	27	84.4	32
Water	29	90.6	32
Waste pit contain sharps	18	100	18

#### Predictors of hand washing

HCWs who had received in-service training on infection control (AOR 2.71, CI: 1.03–7.16), were educated beyond ordinary level (AOR 3.30, CI: 1.44–7.54), mentioned hand washing as one of the infection control measures they knew (AOR 5.70, 2.64–12.32) or those who reported to have acquired a nosocomial infection (AOR 2.84, 1.03–7.84) were more likely to wash their hands than their colleagues without these attributes. The qualification of the HCWs which was significant at bivariate level was not significant at this multivariate level of analysis (Table 4).

**Table 4:** Predictors of hand washing and among healthcare workers in Arua district, 2008

		Hand washing					
Factor	Category	Yes (%)	No (%)	Crude OR (95 % CI)	*P*-Value	Adjusted OR (95 % CI)	*P*-Value
Received in-service training (*n* = 186)	Yes	44 (86)	7 (14)	2.65 (1.06–7.53)	0.036*	2.71 (1.03–7.16)	0.045*
	No	95 (70)	40 (30)	1.00			
Read guidelines (*n* = 185)	Yes	72 (76)	23 (24)	1.14 (0.56–2.33)	0.738	0.71 (0.32–1.59)	0.409
	No	66 (73)	24 (27)	1.00			
Can explain infection control (*n* = 184)	Yes	126 (77)	37 (23)	2.56 (0.87–7.16)	0.060	1.63 (0.54–4.90)	0.379
	No	12 (57)	9 (43)	1.00			
Educated beyond O ‘level (*n* = 186)	Yes	74 (86)	12 (14)	3.32 (1.52–7.59)	0.001*	3.30 (1.44–7.54)	0.005*
	No	65 (65)	35 (35)	1.00			
<35 years (*n* = 186)	Yes	67 (76)	21 (24)	1.15 (0.56–2.37)	0.737	0.68 (0.22–2.07)	0.497
	No	72 (73)	26 (27)	1.00			
Sex (*n* = 186)	Female	78 (77)	23 (23)	1.33 (0.65–2.73)	0.403	0.88 (0.37–2.13)	0.785
	Male	24 (28)	61 (72)	1.00			
Cites hand washing as one of infection control measures known (*n* = 186)	Yes	107 (86)	17 (14)	5.90 (2.73–12.87)	<0.001*	5.70 (2.64–12.32)	0.000*
	No	32 (52)	30 (48)	1.00			
Has ever acquired a nosocomial infection (*n* = 186)	Yes	32 (82)	7 (18)	1.71 (0.67–4.95)	0.302	2.84 (1.03–7.84)	0.043*
	No	107 (73)	40 (27)	1.00			
Qualified beyond nursing assistant (*n* = 186)	Yes	101 (81)	23 (19)	2.77 (1.17–5.13)*	0.004*	1.71 (0.72–4.06)	0.223
	No	38 (61)	24 (39)	1.00			
Healthcare worker from government (public) health facility (*n* = 186)	Yes	86 (78)	24 (22)	1.56 (0.75–3.19)			
	No	53 (70)	23 (30)	1.00	0.058	2.19 (0.96–4.97)	0.061

*statistically significant at *p* = 0.05

## Discussion

We sought to assess the level of implementation of infection control measures in rural Uganda and factors influencing this implementation. We found that most HCWs washed their hands and used gloves when the circumstances required so and that most of them were generally knowledgeable about the different infection control measures. Infection control supplies were generally inadequate, notably soap and personal protective wear. We found that HCWs who had received in-service training on infection control, those that were educated beyond ordinary level, those who cited hand washing as one of the infection control measures, and the ones that reported to have suffered from a nosocomial infection were more likely to wash their hands than their counter parts.

We found a higher level of hand washing than has been reported in several studies [31–34]. This may be probably because most of the observed HCWs were nurses. Numerous studies have shown nurses are more likely to wash their hands compared to doctors and nursing assistants [35–38]. It is also possible that some of the HCWs were aware they were being observed especially since many of them were also interviewed about the same subject later on.

Adequate knowledge was also strongly associated with hand washing. Similarly, Askarian *et al.* found that nurses with adequate knowledge where 14 times more likely to comply with the eight universal precaution measures that the authors assessed [39].

Gloves were the most worn protective gear. This is probably because gloves are relatively affordable compared to other personal protective wear such as gowns. Furthermore, virtually all categories of HCWs need them at some point during their work unlike other specialised wear such as goggles and masks which are more commonly used for non-routine activities such as isolation and major surgeries [40].

The considerably high levels of needle recapping observed at the health facilities (34.4 %) are comparable to those of a cross-sectional study by Sadoh *et al.* where 31.9 % of the HCWs always recapped needles [41]. This finding could be as a result of the lack of clear guidelines on needle recapping. It could also be that many HCWs do not perceive the risks of this practice.

The inadequate number of HCWs who always followed the guidelines may be partially attributed to the low percentage of respondents that had ever read the guidelines. Another reason may be because of the low number HCWs that had ever received in-service training on infection control. Indeed, multivariate analysis showed that HCWs were more likely to wash their hands if they had ever had received training on infection control, a finding that was also observed in the Keren Hospital study [42].

Most of the health facilities had water in at least one of the sections observed. This differed significantly from the service provision survey conducted by the Uganda Ministry of Health and Macro International Inc. which found that only 43 % of the maternal and child health facilities had running water. The same study found that only 56 % of the health facilities had sharps disposal containers compared to 84.4 % of the facilities in this study. These differences may be as a result of the much larger sample size (*n* = 491) for the nationwide survey, regional variations but also because they could been recently procured [24].

Some of the supplies like waste and placenta pits were not available in many facilities probably because they were not that necessary for the level of operations of such facilities or there was limited space to accommodate them. In some situations, some of these facilities were incompatible with the surrounding environment, especially the private clinics in the municipalities.

We found that HCWs who had had prior in-service training were more likely to wash their hands. Indeed, several studies have shown that in-service training enhances compliance with infection control measures [43–46]. Regular training helps to remind the health workers of the importance of these measures.

The results of this study should be interpreted in light of some limitations. It was not possible to observe all sections within each facility which may not have given a comprehensive picture of the infection control compliance. Hence, some observations such as recapping of needles were done by proxy by looking through the waste bins. Staff in a few lower health centres were absent on the data collection days and hence only observations were made in these facilities. We could not verify whether health workers who stated they always followed the infection control guidelines did so due to the limited amount of time of observation. Lastly, some HCWs may have been aware that we were being observed despite measures taken to be discreet, and so this may have resulted in a relatively high level of hand washing compliance found.

## Conclusion

Healthcare workers were fairly knowledgeable about most infection control measures with the exception of isolation and housekeeping. Isolation, the use of personal protective wear and avoiding of needle recapping were inadequately implemented. There were insufficient infection control supplies in most of the health facilities which may have limited the implementation of infection control measures. Healthcare workers are more likely to wash their hands if they have ever received in-service training on infection control, are educated beyond ordinary level, report that they have ever acquired a nosocomial infection or state hand washing as one of the infection control measures they know.

Based on the findings of this study, we recommend that respective health facilities should provide continuous education and training on infection control to all staff. The ministry of health through the district health office should also provide adequate infection control supplies.

## Additional file

Additional file 1:
**Tools used to collect data on infection control implementation in health facilities in Arua District, 2008.**

## Abbreviations

CKN: Christine Kayemba Nalwadda
EB: Esther Buregyeya
FN: Fred Nuwaha
PA: Patrick Anguzu
PW: Peter Wasswa
SNG: Sheba Nacacubo Gitta
