# Supplementary material for: Implementation of infection control in health facilities in Arua district, Uganda: a cross-sectional study
Source: BMC Infect Dis. 2015 Jul 14;15:268. doi: 10.1186/s12879-015-0999-4 (PMC4501062; doi:10.1186/s12879-015-0999-4)
Supplement: Additional file 1: — Tools used to collect data on infection control implementation in health facilities in Arua District, 2008. [file 12879_2015_999_MOESM1_ESM.pdf]

IMPLEMENTATION OF INFECTION CONTROL IN HEALTH FACILITIES IN ARUA DISTRICT, UGANDA: A CROSS-SECTIONAL STUDY

QUESTIONNAIRE

Date………………….
Name of data collector…………………………………………
Name and level of health facility……………………………….
Ward/section……………………………
Personal Details

1. **Age……..**
2. **Sex** Male []
   Female []
3. Marital Status
Married
Single
Widowed
Divorced
4. **Qualification (grade)**
   Nursing assistant []
   Enrolled nurse []
   Registered nurse []
   Enrolled midwife []
   Registered midwife []
   Clinical officer []
   Medical officer []
   Laboratory assistant []
   Other (specify)…………………………
5. **Highest level of education attained**
   Primary []
   Ordinary level []
   Advanced level []
   Tertiary []
6. How many years have you spent in the medical service?

<u>Knowledge on Infection Control</u>

7. Have you ever heard of the term *infection control*? Y/ N
8. What do you understand by the term *infection control*? ……………………………………………………………………………………… ……………………………………………………………………
9. Have you ever heard of infection control guidelines? Y/ N
10. What infection control measures do you know or have heard about? *(tick all that apply)*
   Hand washing []
   Personal Protective Wear []

Safe Injection and Sharps Use []
Safe Medical Waste Disposal []
Isolation []
Disinfection []
Sterilisation []
Housekeeping []
Others (Specify)………………..

11. Of what importance is infection control in the delivery of health services? ………………………………………………………………………………

12. Who should be responsible for ensuring that proper infection control practices are observed? (*tick all that apply*)

Patients []
Patient visitors []
Medical workers []
Administration []
Other………………………………………………………….

13. Have you or any staff acquired infections you think were transmitted by patients attending this facility? Y / N / /Don't Know

14. Have you ever heard of injection/sharps safety? Y/ N

15. If yes, what does it mean?

16. How should used needles be best disposed of? *(Tick all that apply)*

Burn []
Bury []
Incinerate []
Damp in pit latrine []
Damp on skip []

17. What common infections do you think can be transmitted through unsafe needle/sharps handling *(Tick all that apply)*

HIV []
Hep B []
Hep C []
others……………..

18. When is it appropriate for a healthcare worker to wash their hands? *(tick all that apply)*

Before attending to each patient []
After attending to each patient []
Before and after attending to each patient []
Before any surgical/medical procedure []
After contact with dirty surfaces []
After contact with body fluids []
Others……………………………………………………

19. Do you know of any other methods of ensuring adequate hand hygiene besides using soap and water? Y/ N

20. If yes, which are these?

Hand rubs (alcohol, antiseptic) []
Other…………………

21. During what procedures must a healthcare worker wear gloves? *(Tick all that apply)*

Wound dressing []
Blood transfusion []
Surgical operations []
Giving injections []
Obtaining specimen []
Others…………………………………………………………

22. Mention at least 3 diseases you think require mandatory isolation.

a. ……………………………………..
b. …………………………………….
c. …………………………………….

Practice on Infection Control

23. Does this unit/section have a copy of infection control guidelines? Y/ N *(If yes, verify)*

24. If no, why?

25. Have you ever read it? Y/ N

26. How often do you follow the guidelines?

Occasionally []
Often []
Always []
Never []

27. If not always, what prevents you from following them? ………………………………………………………………………………………… ………………………………………………………………………………………… ………………………………………………………..

28. What infection control measures do you routinely practice? *(tick all that apply)*

Hand washing []
Gloves []
Gown wearing []
Disinfection []
Sterilisation []
Housekeeping []
Waste Disposal []
Injection Safety []
Isolation []
Other……………………………………..

29. Have you ever had any training on infection control ever since you started working in this facility? Y / N

30. If yes, when was the most recent one?

2007 []
2006 []
2005 []
2004 []
2003 []
Before 2003 []

31. How often do you wash your hands when dealing with patients?

Always []
Often []
Occasionally []
Never []

32. How often do you use soap when washing your hands?

Always []
Often []
Occasionally []
Never []

33. How often do you wear protective gear?

Always []
Often []
Occasionally []
Never []

34. Which protective gear do you usually put on?

Gloves []
Masks []
Gowns []
Aprons []
Goggles []
Boots []
Others……………………..

35. How often is this section/ward cleaned?

Everyday []
Every 2 Days []
Once a Week []
Twice a week []
Other *(write actual number of days)*…………..

36. What disinfectants do you commonly use to disinfect equipment? *(Tick all that apply)*

Chlorhexidine []
Cetrimide []
Povidone iodine []
Sodium-calcium hypochlorite []
Ethyl alcohol []
Others…………………………

37. What do you use for sterilising surgical equipment?

Autoclave []
Boiler []

38. Do you have separate bins for sharps and infectious medical waste? Y/ N (*If yes, verify)*

39. Do you recap needles after use? Y/ N

40. Do you have an isolation unit in this facility? Y/ N (*if yes, verify)*

Attitude

41. Do you think it is necessary to observe infection control practices in all circumstances when handling patients? Y/ N

42. In your opinion, do you think it is practical to observe all the recommended infection control practices as stated in the guidelines? Y/ N

43. If no, why? ---

44. In your opinion, what is the most important infection control practice that needs to be observed? *(tick only one)*

Hand washing []
Personal Protective Wear []
Safe Injection and Sharps Use []
Safe Medical Waste Disposal []
Isolation []
Disinfection []
Sterilisation []
Housekeeping []
Others (Specify………………………..

45. Are there any infection control measures you find particularly difficult to observe? Y/ N

46. If yes, what practices are these? *(tick all that apply)*

Hand washing []
PPE []
Disinfection []
Sterilisation []
Housekeeping []
Isolation []
Other………………………………………………

47. Why is this so?...(*tick all the apply*)

48. What diseases you particularly concerned of being at a high risk of acquiring from the patients you are attending to?

HIV []
Hep B []
Hep C []
TB []
Pneumonia []
Skin infections []
others……………..

49. What advice would you give to all those responsible on how to improve infection control in this health facility? ---------------------------------------

KEY INFORMANT GUIDE

Date………………………

Name and level of health facility……………………………

Personal details

Name of key informant……..

Age……………… Sex M []

F []

Position of responsibility…………………………………………

Level of training (qualification)…………………………………….

1. How would you describe the infection control situation in this health unit and do you think it is adequate?
2. What mechanisms do you have in place to ensure that infection control measures are implemented in this health unit?
3. How is this facility able to monitor hospital-acquired infections? (ask about post-operative surgical infections and injection abscesses)
4. Could you explain the mechanisms you have in place to monitor the use of antibiotics?
5. How often do you get personnel from the HSD or District Health Office to monitor or supervise infection control activities? (Ask when the last supervision was held, the method and promptness of feedback)
6. What structures does this facility have to educate patients, their visitors or staff about infection control?
7. What stock-outs of supplies and equipment needed for infection control do you normally experience? (probe which supplies are most commonly out of stock, how often this occurs and how long these stock-outs last)
8. How do the current staffing levels of this health facility compare with the Ministry of Health requirements?
9. What advice would you give to the higher authorities on how to improve infection control in this health facility?

INFECTION PREVENTION AND CONTROL SUPPLIES CHECKLIST

**A. Hand Hygiene**	**Available** *(tick and add comment where indicated in specified section)*	**Not available** *(tick and add comment where indicated in specified section)*
Soap at washing points		
Tap/ vessel for pouring water (tank, borehole or protected spring) *NB. Confirm if water is flowing from source*		
Alcohol-based hand solutions		
Antiseptics e.g. dettol		
**B. Personal Protective Equipment**		
Disposable gloves		
Protective eye wear		
Face masks		
Lab coats/gowns/ aprons		
**C. Injection Safety/ Sharps Management**		
Single-use injections		
Sharps disposal containers *(Puncture proof and water resistant)*		
Sharps injury log		
**D. Disinfection/ Sterilization**		
Functional Autoclave *(NB* ***not*** *boiler, regularly serviced and with spares, temp. and pressure gauge working)*		
Test-strips for autoclaves		
Suitable disinfectant		
**E. Waste disposal**	**Available**	**Not available**
color-coded plastics bags/containers or other sharps disposal vessels		
containers with secure lids		
Safety signs for hazardous wastes on containers		
Strong gloves for waste disposal		
Functional incinerator		
Waste pit for non-infectious waste (*not overfilled*)		
Functional placenta pit within premises		

Deep pit for burying sharps/blades/injections		
**F. Housekeeping items** *(either in the store or on site)*	**Available**	**Not available**
Cleaning items *(ignore if service is tendered)*<br>▪ Brooms<br>▪ Rags<br>▪ Brushes<br>▪ Buckets<br>▪ Gloves		
Disinfectants *(indicate type)*		
Clean and well kept compound		
Pit latrine within premises of h/ facility *(tick all that apply)*<br>▪ Functional<br>▪ Clean<br>▪ Soap available<br>▪ Water available		

OBSERVATION CHECKLIST

**Hand Hygiene**	**Yes**<br>*(Add comment where indicated in the specified section)*	**No**<br>*(Add comment where indicated in the specified section)*
Does healthcare worker wash hands with soap and water before and/or after:<br>• Before touching patient?<br>• After touching patient?<br>• Wound dressing, suturing, catheterization, injections, norplant insertion or removal, intravenous infusion or dental examination,<br>• Touching patient surroundings?<br>• Touching any bodily fluids?		
**Personal Protective Equipment**		
Does healthcare worker wear gloves before wound dressing, suturing, insertion or removal of norplant, IV infusion or dental examination?		
Are physicians and nurses or other direct service providers wearing laboratory coats?		
**Sharps management**		
Do healthcare workers recap used needles?		
**House Keeping**		
Are the sections below clean? *(dust and litter free, well-mopped)*<br>▪ OPD<br>▪ maternity,<br>▪ injection and dressing rooms		
